# Endodontic management of open apex using MTA and platelet – rich fibrin membrane barrier: A newer matrix concept

**DOI:** 10.4317/jced.51178

**Published:** 2013-12-01

**Authors:** Ambica Khetarpal, Sarika Chaudhry, Sangeeta Talwar, Mahesh Verma

**Affiliations:** 1 Senior Research Associate. Department of Conservative dentistry and Endodontics, Maulana Azad Institute of Dental Sciences, New Delhi; 2 Associate Professor. Department of Conservative dentistry and Endodontics, Maulana Azad Institute of Dental Sciences, New Delhi; 3 Professor and Head. Department of Conservative dentistry and Endodontics, Maulana Azad Institute of Dental Sciences, New Delhi; 4 Professor and Head. Department of Prosthodontics, Maulana Azad Institute of Dental Sciences, New Delhi

## Abstract

Objectives: Endodontic management of open apex using MTA and platelet – rich fibrin membrane as an apical matrix barrier. Study design: An immature tooth with pulpal necrosis and periapical pathology imposes a great difficulty to the endodontist. Endodontic treatment options for such teeth consist of conventional apexification procedure with and without apical barriers. This article demonstrates the use of an apical matrix barrier in form of a platelet rich fibrin membrane for stabilization of MTA in root end apexification procedure. PRF is an autologous fibrin matrix containing a large quantity of platelet and leukocyte cytokines, which enhance healing by release of growth factors. These case reports present apexification and successful healing with combined use of MTA and PRF membrane as an apical barrier Results: PRF membrane can serve as an efficient apical matrix for condensation of MTA. Combination of PRF membrane and MTA is an effective method for management of difficult cases of open apex. PRF is a strong fibrin membrane enriched with platelet and growth factors that accelerate periapical healing.

** Key words:**Apexification, apical barrier, platelet rich fibrin (PRF), mineral trioxide (MTA).

## Introduction

MTA has been shown to be a very effective root filling material for sealing immature root canals with open apices that could otherwise impose technical challenges in obtaining adequate obturation. MTA has an ability to facilitate periradicular healing by inducing hard-tissue formation (1). But in some cases with wide open apices, adequate condensation of MTA is difficult to achieve as the material may get extruded beyond the apex. Therefore an apical matrix is used for the controlled placement of MTA to a desired level. Various biocompatible materials have been tried as apical matrix in the past. These include: tricalcium phosphate (2), collagen calcium phosphate (3), osteogenic protein-1, bone growth factor and oxidized cellulose (4), proplast, (a polytetrafluor-ethylene and carbon felt-like porous material) (5), barium hydroxide (6), true bovine bone ceramics, and dentin chips (7). “Modified matrix concept” for repair of perforation utilized resorbable collagen as a matrix followed by condensation of MTA (8).

Platelet-rich fibrin (PRF) developed in France by Choukroun and Dohan (9) represents a new step in the platelet gel therapeutic concept. PRF is a matrix of autologous fibrin, in which are embedded a large quantity of platelet and leukocyte cytokines during centrifugation. PRF obtained from Choukroun’s technique is a strong fibrin membrane enriched with platelet and growth factors. The easily applied PRF membrane serves as a matrix to accelerate the healing of wound edges (10).

Therefore, present case reports highlight the nonsurgical management of symptomatic teeth with immature apices and large periapical radiolucencies using PRF membrane matrix and MTA to promote periapical healing.

## Case Report

- Case Report 1 

**Figure 1:**
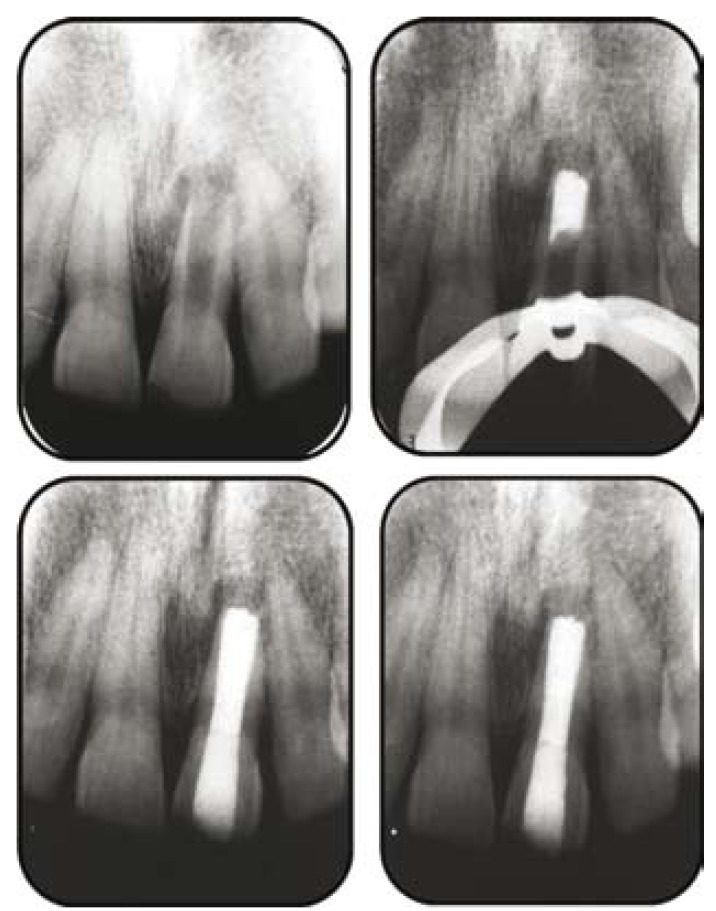
a) Diagnostic radiograph; b) MTA plug; c) Post obturation radiograph; d) Healing evident after 3 months.

A 15-year-old male patient reported with a chief complaint of a discolored maxillary right central incisor. History revealed that the patient had suffered trauma at the age of 8 years. Radiographic examination revealed an immature tooth with a wide open apex and a radiolucent area in proximity of the apex of the tooth (Fig. 1). Endodontic access opening was done under local anesthesia , and a periapical radiograph was taken to determine the working length. The root canal was lightly cleaned with a hand file under irrigation with 1.3% NaOCl. The root canal was then dried with sterile paper points. Calcium hydroxide was placed in the root canal, and the patient was recalled after one week. One week later, the tooth was again isolated under rubber dam, the calcium hydroxide dressing was removed by hand instrumentation, and irrigation was done with 1.3% NaOCl and 17% liquid EDTA Smear Clear (SybronEndo, CA, USA). The root canal was then dried with sterile paper points.

**Figure 2:**
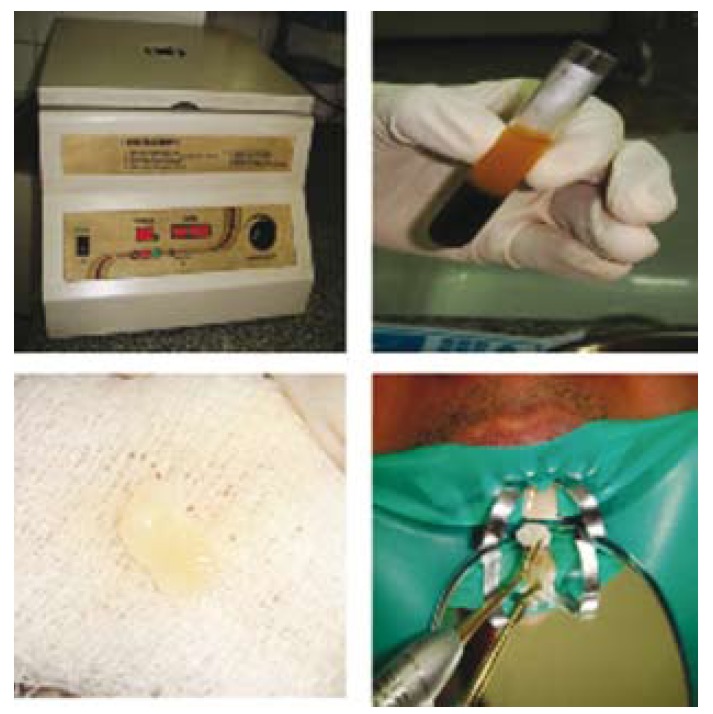
a) Centrifuge; b) 3 layers: top is platelet poor plasma, middle is PRF, and bottom layer contains red blood cells (RBC’s); c) PRF membrane in gauze; d) Carrying PRF membrane in the canal to form matrix.

PRF membrane was used in the formation of the artificial barrier. Patient’s whole blood was drawn into 10 ml glass coated plastic tubes using PRF collection kit without anticoagulant and immediately centrifuged in Process® centrifuge (PC-02, Process Ltd., Nice, France) (Fig. 2) at 3000rpm for 10 minutes. Three layers got formed in the tube (Fig. 2): a base of RBCs, at the bottom, acellular plasma on the surface, and PRF clot in the middle. The fibrin clot was easily separated from the lower part of the centrifuged blood. The PRF clot was gently pressed into a membrane with a sterile dry gauge (11) (Fig. 2).

The membrane was packed against the bone and was pushed beyond the apex into the bony space formed due to the periapical lesion to form a matrix for the placement of MTA (Fig. 2).

A thick mixture of White Proroot MTA (Dentsply, Switzerland) was then prepared and applied to the apical portion of the canal using a small plugger and the butt end of sterile paper points (Fig. 1) and excess material was cleared from the walls. Moistened gauze was placed in the remainder of the canal and the access cavity sealed using glass ionomer cement (Fuji, GC Corporation, Tokyo, Japan). Gutta percha backfill was performed using Obtura (Obtura/Spartan, Fenton, MO, USA), and the access cavity was sealed using composite resin (Fig. 1) A radiograph confirmed the completion of the endodontic therapy.

A 3-month follow- up revealed complete periapical healing and bone formation (Fig. 1).

The clinical follow-up at one year showed the patient functioning well with no reportable clinical symptoms and an absence of any sinus tract formation. The radiographic follow-up at one year showed complete healing of the periapical radiolucency and regeneration of the periradicular tissues.

- Case Report 2 

**Figure 3:**
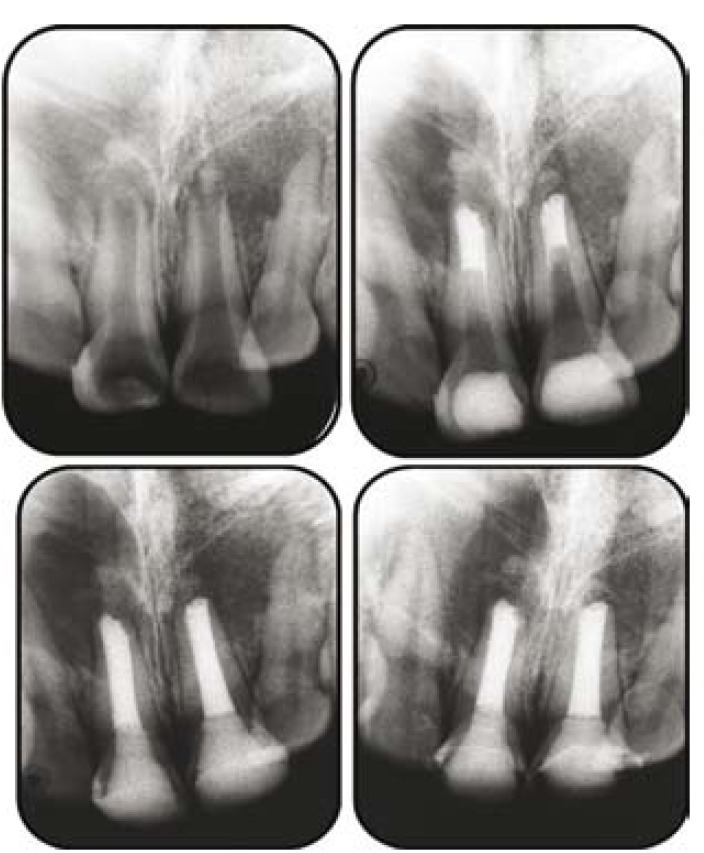
a) Preoperative radiograph; b) MTA plug; c) Post obturation radiograph; d) Healing eident after 3 months.

A 17-year-old woman sought treatment for pain and swelling in the maxillary anterior region. Both the central incisors were discoloured and had severe tenderness to percussion. IOPA Xray (Fig. 3) revealed open apices and large periapical radiolucency in relation to these teeth.Although surgical removal of the periapical lesion was also an available option, nonsurgical treatment was opted considering the age and amount of trauma expected during surgical treatment. Endodontic therapy was started and thorough biomechanical preparation was done using involving circumferential filling with a size 80 K file (Dentsply, India). Thereafter, calcium hydroxide was placed in canal and patient was recalled after 2 weeks. Patient was asymptomatic at the recall appointment. The medicament was removed from the canal followed by irrigation with 1.25% sodium hypochlorite. The apical matrix/barrier was created by carrying PRF membrane through the canal using hand pluggers (Dentsply, India) and packing it in periapical area (Fig. 3). This was followed by a placement of 5 mm apical plug of white PROROOT MTA (MTA ™ Dentsply, India) using a finger plugger. The patient was asymptomatic at 1-week recall visit. Therefore, remaining canal was obturated using resin-based endodontic sealer (AH 26, Dentsply India) and thermoplasticized gutta percha (Obtura II, J. Morita Corporation, Japan) (Fig. 3). The 9-month follow-up radiograph of the patient showed reduction in the size of the periapical lesion (Fig. 3).

## Discussion

The major problem in cases of a wide open apex is the need to limit the material, thus avoiding the extrusion of a large amount of material into the periodontal tissue.

Using a matrix avoids the extrusion of the material into the periodontal tissues, reduces leakage in the sealing material and allows favorable response of the periodontal tissues.

The apical barrier technique utilizing calcium sulfate or a combination of calcium sulfate and collagen in a powdered form has been performed in the past. Various materials have been used for formation of apical barrier during apexification. This case report has introduced a new concept of using PRF as an apical matrix membrane.

PRF is a matrix of autologous fibrin, in which are embedded a large quantity of platelet and leukocyte cytokines during centrifugation (9). The intrinsic incorporation of cytokines within the fibrin mesh allows for their progressive release over time (7-11 days), as the network of fibrin disintegrates.(11) The easily applied PRF membrane acts much like a fibrin bandage serving as a matrix to accelerate the healing of wound edges (13).

According to Simonpieri et al (2009) (14), the use of this platelet and immune concentrate offers the following 4 advantages: First, the fibrin clot plays an important mechanical role, with the PRF membrane maintaining and protecting the grafted biomaterials and PRF fragments serving as biological connectors between bone particles. Second, the integration of this fibrin network into the regenerative site facilitates cellular migration, particularly for endothelial cells necessary for the neo-angiogenesis (9), vascularization and survival of the graft. Third, the platelet cytokines (PDGF, TGF- IGF-1) are gradually released as the fibrin matrix is resorbed, thus creating a perpetual process of healing (15). Lastly, the presence of leukocytes and cytokines in the fibrin network can play a significant role in the self-regulation of inflammatory and infectious phenomena within the grafted material.

The combination of PRF membrane as a matrix and MTA has been demonstrated to be an effective alternative for creating artificial root-end barriers and to induce faster periapical healing for single visit apexification of the cases with large periapical lesions. The precise mechanism of action of PRF is yet to be proved. PRF is a second generation platelet concentrate which is still under study and many more advancements in its clinical applications are expected in near future.
